# Associations Between Diabetes, Diabetes Duration, and Psychiatric Difficulties: A Nationwide Cross-Sectional Study

**DOI:** 10.1192/bjo.2026.11071

**Published:** 2026-06-30

**Authors:** Jong-Hyun Jeong, Sang-Yeol Lee, Bo-Hyun Yoon, Hyung Mo Sung

**Affiliations:** 1 St. Vincent’s Hospital, The Catholic University of Korea, Suwon, Korea, Republic of; 2 College of Medicine, Wonkwang University, IKsan, Korea, Republic of; 3 Naju National Hospital, Naju, Korea, Republic of; 4 Soonchunhyang University Gumi Hospital, Gumi, Korea, Republic of

## Abstract

**Aims:**

To investigate relationships between diabetes, diabetes duration, and psychiatric difficulties using data from a nationwide survey and to examine associations between psychiatric difficulties and physician diagnosis, treatment status, and glycaemic control in diabetes patients by performing subgroup analyses.

**Methods:**

Data from the 2022 Korea National Health and Nutrition Examination Survey were analysed. A representative sample of 4,475 adults aged 19 years or older was obtained using stratified, multistage, clustered sampling. Psychiatric difficulties were assessed using PHQ-9 for depression, GAD-7 for anxiety, and face-to-face interviews for suicidality. Diabetes patients were categorized by diabetes duration (<5, 5−9, 10−14, and ≥15 years). Statistical models were adjusted for demographic and clinical covariates.

**Results:**

Diabetes patients were significantly more likely to experience depression than controls (aOR=1.818). Those with a diabetes duration of ≥15 years showed significant associations with depression (aOR=2.830), suicidal ideation (aOR=2.496), and suicidal plans (aOR=4.604). Such associations were not found in shorter duration groups. Subgroup analyses revealed links between suicidal ideation and being diagnosed by a physician or receiving treatment. No significant associations were found between glycaemic control and psychiatric difficulties.

**Conclusion:**

Depression was more prevalent in diabetes patients than in controls. A diabetes duration of ≥15 years was significantly linked to higher rates of depression and suicidality. Routine psychiatric screenings after 15 years of diabetes duration could enhance public health strategies. Addressing societal stigma and alleviating burdens of diabetes management might improve mental health outcomes.

